# What Saves the Day?: Identifying Protective Factors That Buffer the Negative Effects of Daily Stressors

**DOI:** 10.1093/geroni/igaa057.2185

**Published:** 2020-12-16

**Authors:** Joanna Hong, Meng Huo, Christiane Hoppmann

## Abstract

Older adults frequently experience daily stressors and are at increased risk of adverse health consequences. Emerging studies have focused on factors that may buffer older adults’ well-being and strengthen their resilience to stress. This symposium adds to this burgeoning literature and presents studies that identify a wide range of such stress-buffering factors, including individual characteristics and daily social and emotional experiences. Huo et al. considered older adults’ empathy and found that more empathic individuals tended to use constructive strategies but not destructive strategies when coping with interpersonal tensions. When daily tensions occurred, more empathic individuals were also better at maintaining their mood. Kim et al. showed that older adults’ marital status influenced their irritable encounters with grown children. Although irritable encounters were associated with increased daily negative mood, this link varied by parents’ marital status. Hong et al. assessed the link between daily stressors and negative affect among older adults with varying levels of loneliness. They found that positive interpersonal encounters were particularly protective for lonely older adults’ daily affect. Leger et al. further examined the buffering effects of trait level and same-day positive emotions during times of stress. Higher trait and same-day levels of positive emotions reduced negative emotions on the day following a stressor. Together, findings might inform future interventions aimed at increasing the daily experience of older adults. Dr. Hoppmann will serve as the Discussant and summarize the theoretical and methodological contributions of these studies. She will discuss challenges in this field and directions for future research.

